# Diversity of gut microbiome in Rocky Mountainsnail across its native range

**DOI:** 10.1371/journal.pone.0290292

**Published:** 2023-11-27

**Authors:** Bridget N. Chalifour, Leanne E. Elder, Jingchun Li

**Affiliations:** 1 Department of Ecology and Evolutionary Biology, University of Colorado Boulder, Boulder, Colorado, United States of America; 2 Museum of Natural History, University of Colorado Boulder, Boulder, Colorado, United States of America; National Cheng Kung University, TAIWAN

## Abstract

The animal gut microbiome is often a key requirement for host nutrition, digestion, and immunity, and can shift in relation to host geography and environmental factors. However, ecological drivers of microbiome community assembly across large geographic ranges have rarely been examined in invertebrates. *Oreohelix strigosa* (Rocky Mountainsnail) is a widespread land snail found in heterogeneous environments across the mountainous western United States. It is ideally suited for biogeography studies due to its broad distribution, low migration, and low likelihood of passive transport via other animals. This study aims to uncover large-scale geographic shifts in the composition of *O*. *strigosa* gut microbiomes by using 16S rRNA gene sequencing on samples from across its native range. Additionally, we elucidate smaller-scale microbiome variation using samples collected only within Colorado. Results show that gut microbiomes vary significantly across broad geographic ranges. Several possible ecological drivers, including soil and vegetation composition, habitat complexity, habitat type, and human impact, collectively explained 27% of the variation across Coloradan *O*. *strigosa* gut microbiomes. Snail gut microbiomes show more similarity to vegetation than soil microbiomes. Gut microbial richness was highest in the rocky habitats and increased significantly in the most disturbed habitats (low complexity, high human impact), potentially indicating signs of dysbiosis in the snails’ gut microbiomes. These small-scale environmental factors may be driving changes in *O*. *strigosa* gut microbiome composition seen across large-scale geography. This knowledge will also help us better understand how microbial associations influence species survival in diverse environments and aid wildlife conservation efforts.

## Introduction

Microbiological research has revealed a glimpse of the integral, yet largely unknown role that microorganisms play in many diverse animal taxa [1]. The microbiome is an important adaptation in many animal species, and often a key requirement for host health [2–6]. The gut microbiome is of particular importance to animals, as it aids in host digestion, nutrition, and immunity [2, 5–7]. While it is widely recognized that factors like host phylogeny, behavioral patterns, habitat composition, diet, and geographic location can influence microbiome composition, there is a gap in knowledge in understanding the relative importance of these factors [3, 8–10]. A comprehensive understanding of microbiome compositions in wild animals requires teasing apart the interacting effects of geography and related environmental factors.

One of the most striking factors driving microbiome changes intraspecifcally is host geography [10]. Many animal gut microbiomes are dynamic, and shift depending on geography. For example, the endangered takahē bird’s gut microbiome, approximated by using fecal samples, shows significant variation by geographic location [11]. Wild house mice gut microbiomes are also significantly tied to trapping location [12]. However, not all species show a microbiome response to geography. In the Atlantic salmon, the gut microbiome does not vary significantly across a geographic gradient, and there was no discernable effect of locality, either by country or origin or specific study site [13]. Instead, the gut microbiome responds more strongly to other life history factors, like life-cycle stage [13]. Likewise, woodrats microbiome structure is governed more strongly by host phylogeny over geography [14].

Geographic differences may be an indirect cause of microbial community shifts, with the possible direct causes covarying with location [10]. In many animal systems, the gut microbiome is shown to fluctuate based on shifts in ecological factors, such as diet composition and habitat fragmentation, which are often interrelated to geographic differences [10, 15–17]. Fluctuations in microbiome composition may also represent adaptations to abiotic challenges tied to location differences. For example, gut microbiome changes in macaques were attributed to adaptions to high-altitude environments [18]. Therefore, it is necessary to also investigate other possible drivers of variation that covary with geographic location.

Despite the importance of microbiome geography, many animal microbiome studies still only focus on a snapshot of the species’ microbiome, rather than representatives from varying habitats and environments encompassing its native range. It is imperative to understand how microbiomes vary over broad spatial gradients, as changes in the microbiome can impact host health and potentially cause dysbiosis [11, 18]. Additionally, investigating intraspecific microbiome shifts is a useful tool for understanding the life history of a species. Research investigating microbiome differences in a single species over its entire range can inform conservationists in protecting population subsets that are threatened or endangered only in certain geographic areas, and in breeding captive individuals that can be reintroduced successfully [17].

**Fig 1:**
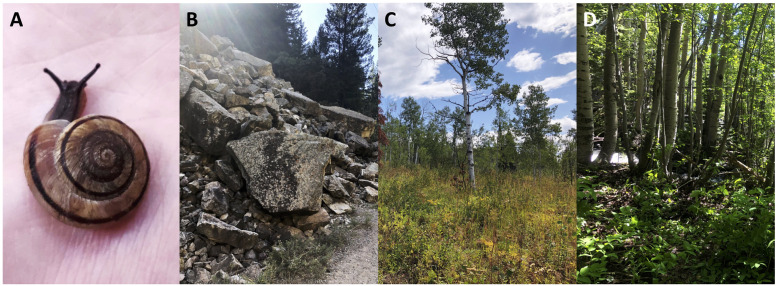
**(A)** A live specimen of *Oreohelix strigosa*, as found in a various habitat types including **(B)** rocky, talus slopes (Jess Weaver Trail, Glenwood Springs, CO); **(C)** tall grasslands (Lower Bear Trail, Routt, CO); and **(D)** lush forests (Mountain Research Station, Ward, CO). All photos by B. Chalifour.

In order to investigate biogeographic influence on the gut microbiome, the ideal model organism is a species with an extensive geographic range, a low dispersal rate—allowing for increased ecological specialization, a proven presence of a gut microbiome, and abundance in museum collections. *Oreohelix strigosa* (Rocky Mountainsnail, Fig 1A) is a widespread land snail species ranging across the mountainous western United States. *O*. *strigosa* is found in a variety of environments, including dry Southwestern habitats and the near-alpine of the Rocky Mountains. It is also found across gradients of other habitat-shaping factors, like levels of human disturbance and habitat complexity. It is ideally suited for biogeography studies due to its broad distribution, low migration, and low likelihood of passive transport via other animals.

Geographic differences have already been shown to shape other life history parameters of *Oreohelix* species. For instance, shell ornamentation is tied to geologic factors like the availability of calcium carbonate [19]. Coloradan *O*. *strigosa* have also been shown to contain a diverse, but stable gut microbiome [20], and the species is well documented from a broad range of geographic origins in museum collections nationwide. However, the composition and diversity of the microbial communities within *O*. *strigosa* across its native range has not been yet characterized. This research aims to uncover shifts in the composition of *O*. *strigosa* gut microbiomes in a wide geographic range, and to elucidate smaller-scale microbiome variation in response to environmental factors across the Colorado Front Range.

In this study, we strive to answer the following questions: 1) Is there an association between location and *O*. *strigosa* gut microbiome diversity? 2) If there is, what ecological aspects within locations are associated with changes in microbiome composition? We collected snail gut samples encompassing much of *O*. *strigosa’s* native range, to determine what, if any, microbiome patterns persist at a broad, geographic scale. To narrow in on what shifts may be happening between locations and why, we also field-collected *O*. *strigosa* from localities across the Colorado Front Range along with corresponding environmental metadata.

## Materials and methods

**Fig 2:**
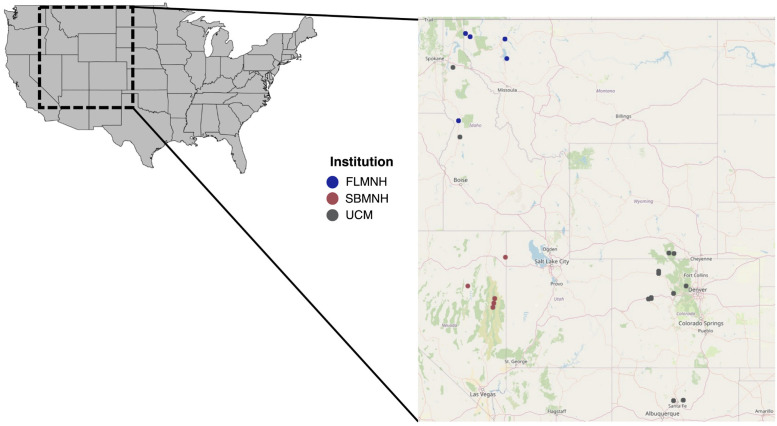
Map of the Rocky Mountain region of the United States, with collection points across the native range of *Oreohelix strigosa* and their corresponding institution of origin indicated: SBMNH (Santa Barbara Museum of Natural History), UCM (University of Colorado Museum of Natural History), or FMNH (Florida Museum of Natural History).

This study consists of data from 151 snails collected across 10 localities (Fig 2) across the Colorado Front Range from the summer of 2019, along with 30 soil samples and 30 vegetation samples from those same 10 localities (three samples per locality for soil and vegetation). We also used data taken from 93 ethanol-preserved snails collected across states found in *O*. *strigosa’s* native range, including Idaho, Montana, New Mexico, Wyoming, and Utah (Fig 2), which were loaned from three natural history museum collections (University of Colorado Boulder Museum of Natural History [UCM], Florida Museum of Natural History [FMNH], and Santa Barbara Museum of Natural History [SBMNH]).

### Museum sample collection

*Oreoheix strigosa* collected across its native range within the last decade were sampled from museum specimens. Samples were loaned and shipped to UCM, where gut DNA extractions occurred in accordance with their home museums’ destructive sampling policies. All museum samples were stored under the same conditions—80–95% ethanol—which are ideal conditions to conduct DNA/RNA analyses for museum samples. While many were preserved for differing lengths of time (a range of 0–16 years), previous work comparing *O*. *strigosa* musem-preserved samples over a longer storage duration (up to 98 years) shows that storage time does not impact the gut microbiome enough to justify adjusting for batch effects based on storage time [21]. A total of 24 specimens from the SBMNH were used in this study, originally collected from Nevada. A total of 29 specimens were used from the FMNH, collected from locations in Idaho (8 specimens) and Montana (21). A total of 40 specimens were used from the UCM, collected from locations in Idaho (11 specimens), New Mexico (13), and Wyoming (16). A complete list of specimens used can be found in S1 Table.

### Field sample collection

#### Collection site habitat assessment

Sampling sites from the Colorado Front Range were determined based on a combination of optimal environmental conditions for *O*. *strigosa* habitat, and a variety of anthropogenic disturbance factors known to influence snail grazing. Metadata taken at each site included GPS coordinates, temperature and humidity readings, the dominant habitat “type” (talus rocky slope, hereafter: rocky; grassland; and forest; Fig 1B–1D) along with qualitative microhabitat observations including a score of habitat complexity and human impact. Habitat complexity was based on the presence of a water body, topography, vegetation, and exposed rock. These parameters are based on the biotic and abiotic requirements of land snails and are each assigned a numeric value from 0–2 based on the level of the habitat feature at each site [22]. The summed values serve as a single total value to quantify the level of habitat complexity, and were then assigned a single categorical as “High” and "Low” complexities, scores of less than 7 were designated as “Low”, and 7 and higher were “High” (Table 1). Human impact was given a single categorical value of “Low”, “Medium”, or “High” (Table 1) based on measures of population density, impervious surface percentage, and Human Built-Up and Settlement Extent (HBASE). Sites designated as “Low” show no HBASE, no impervious surface coverage, and lowest human population density (<1 persons/ sq. km or no data) and were often federally protected. Sites designated as “Medium” included some Hanging Lake sites, which are federally protected, but the trail has been heavily trafficked by visitors in recent years and were therefore more disturbed than other protected sites. Sites designated as “High” are in HBASE areas, have a larger population density, and have a higher percentage of impervious surface coverage; none of these sites were federally protected. These parameters were measured using the Global Man-made Impervious Surface (GMIS) data from Landsat v1, a tool which provides high spatial resolution (to 30 meters) estimates of global man-made imperviousness [23]. We also used colloquial knowledge and physical observations of land use by people to make judgments on the levels of human impact in each site.

**Table 1:** Relevant metadata from Colorado *O*. *strigosa* snail population localities.

Locality Name	Sample Size	Habitat Complexity	Human Impact	Habitat Type
Mountain Research Station (MRS)	18	High	Low	Forest
Jess Weaver	13	High	Low	Rocky
Glenwood Canyon	15	Low	High	Rocky
Hanging Lake Site 1	13	High	Medium	Forest
Hanging Lake Site 2	19	High	Low	Forest
Hanging Lake Site 3	13	High	Medium	Forest
Lower Bear Trail	12	Low	Low	Grassland
Steamboat Springs	26	Low	High	Grassland

#### Snail collection

In the summer of 2019, between June and September, when terrestrial snails of the Rockies are most active, we collected fresh, living samples of *Oreohelix strigosa* from eight locations across the Colorado Front Range (Table 1). These included populations from the University of Colorado Mountain Research Station; Jess Weaver trail and three locations along the Hanging Lake trail, all in White River National Forest; Lower Bear Trail in Routt National Forest; Steamboat Springs; and Glenwood Canyon. We used a qualitative collection method to collect specimens for this study, in accordance with [20, 21]. All collections were taken with the appropriate permitting for invertebrates, along with special permissions from private landowners, the University of Colorado Mountain Research Station, White River National Forest, and Routt National Forest.

Snails were first drowned in distilled water and preserved in 95% ethanol for 24 hours, then transferred to and kept in 80% ethanol for permanent preservation as they were extracted, in accordance with UCM policies.

#### Soil and vegetation collection

Six surface soil cores were taken from 0-10cm in depth within each collection site that displayed the dominant ecosystem vegetation type (below where snails were collected), and stored in sterile WhirlPak bags. We selected the sampling range of 0–10 cm for many reasons. First, this is a commonly used depth in comparable soil microbiome studies [24]. Second, most of the belowground microbial biomass is concentrated in the top 10 cm [24]. Finally, *O*. *strigosa* typically burrows within this top layer of the soil, and we hypothesized that most of its environmentally augmented microbiome would come from contacting with this soil layer. The soil cores were sieved to 2mm to remove larger litter fragments and become homogenized.

Fresh and dried vegetation was taken from areas directly adjacent to snail populations at each site and stored in sterile WhirlPak bags. Like soil samples, 3–4 vegetation samples were taken at each collection site and homogenized.

### Microbial DNA extraction and microbiome analysis

All dissections were performed aseptically and snail gut tissues were collected following protocols in [20].

Snail gut genomic DNA was extracted using the E.Z.N.A. Mollusk DNA Extraction kit (Omega Bio-Tek, Norcross, GA, USA) according to the manufacturer’s instructions, with the inclusion of extraction blanks as a negative control. DNA was extracted from 0.25g of each soil and vegetation sample using the PowerSoil DNA isolation kit (Qiagen) according to the manufacturer’s instructions, also including extraction blanks as a negative control. The 16S rRNA gene was amplified using the 515F/806R primers modified to include Illumina adapters and barcodes [25]. Library preparation and sequencing was conducted by the Center for Microbial Exploration at the University of Colorado Boulder. DNA was pooled, normalized with the SequalPrep normalization plate kit (Invitrogen, Carlsbad, CA, USA), and then sequenced in one run on the Illumina MiSeq platform PE300 (Illumina Corporation, San Diego, CA, USA) using a 2-by-150-bp paired end chemistry with the MiSeq V2 300-cycle kit (Illumina, San Diego, CA, USA). Amplicon reads were demultiplexed using the open source “idemp” tool (https://github.com/yhwu/idemp), and adapters were cut from the sequences using the open source “cutadapt” tool (https://cutadapt.readthedocs.io/en/stable/; version 1.8.1) with default parameters and “—minimum-length” set at 50. Sequences were then quality filtered (parameters maxEE = 2, truncQ = 2, maxN = 0), trimmed (150bp) and merged using the DADA2 pipeline (version 1.14.1) [26] to then infer amplicon sequence variants (ASVs) using the Silva reference database (version 138.1 SSU Ref NR 99) and remove chimeras. Additionally, eukaryote, chloroplast, and mitochondrial sequences were removed from the sequence data set. Taxonomic filtering to remove rare taxa (<5 reads) was performed using the {mctoolsr} R package (https://github.com/leffj/mctoolsr/). Negative extraction and PCR blanks had significantly fewer sequences than gut, vegetation, and soil samples, any sequences in negative controls were analyzed taxonomically and found to not fall into the same taxonomic groupings as the prevalent strains found in non-control samples. We chose to not rarefy our samples to one value as the project combined samples with significantly different read counts caused by a difference in sample type—for example, soil versus snail gut have drastically different average read counts. Additionally, relative abundances of taxa were not transformed, instead, we employed statistical methods designed to accommodate these data.

### Species identification

Snail species identification was confirmed using the COI mitochondrial gene amplified using primer sets LCOI490 5’-GGTCAACAAATCATAAAGATATTGG-3’ and HCO2198 5’-TAAACTTCAGGGTGACCAAAAAATC-3’ to compare against the most up-to-date *Oreohelix* COI molecular phylogeny from [19]. PCR amplifications were performed in a total reaction volume of 26 *μ*L with 12.5 *μ*L GoTaq Green Master Mix (Promega), 10.5 *μ*L of nuclease-free water, 1 *μ*L of each primer and 1 *μ*L of the DNA template. The PCR protocol for COI included an initial denaturation at 96 °C for 2 minutes, 9 cycles of 96 °C for 40 s, 59 °C for 60 s and 72 °C for 60 s, 37 cycles of 96 °C for 40 s, 46 °C for 60 s and 72 °C for 60 s, and a final extension at 72 °C for 7 minutes. PCR products were assessed through gel electrophoresis. Amplified products were sequenced by Sanger sequencing at Quintara Biosciences (California). Sequences were compared against a database of the published sequences from [19] using the command-line version of NCBI BLAST (version 2.2.18) [27]. All snails used in this study were confirmed to be *O*. *strigosa*.

### Statistical analyses

Data analysis was completed using R statistical software (version 4.2.0) [28]. We examined gut microbiome community composition differences among our major treatment groups (i.e., explanatory variables) across all snails sampled, including the state collected and verbatim locality collected, along with several other ecological factors only in the Colorado-collected samples, including habitat type, habitat complexity, and human impact.

Snail gut microbial compositional differences were assessed using a non-metric multidimensional scaling analysis (NMDS) based on location collected (both state and verbatim locality), habitat type, habitat complexity, and human impact. We used microbial community diversity as the dependent variable for each explanatory variable. We used a permutation analysis of variance (PERMANOVA) to test for significant differences in microbial compositions univariately among different explanatory metadata variables (“adonis2” function in {vegan} package, https://cran.r-project.org/web/packages/vegan/index.html).

We ran a Mantel test in the {vegan} and {geosphere}(project.org/package = geosphere) R packages to examine if there was correlation between snail population geographic distance (using latitude/longitude coordinates) and microbial community similarity (Bray-Curtis dissimilarity). We used the {leaflet} R package [29] to plot collection points and originating institutions in Fig 2.

Within Colorado-collected samples, we looked at taxonomic differences between sample types (snail gut, associated soils, and associated vegetation) and conducted a Venn analysis of the ASVs associated to each sample type. We then examined how microbial richness was affected by sample type, and also investigated species evenness and Shannon index as factors of microbial richness.

We used the “return_top_taxa” function of the {MCToolsR} package to initially discern which taxa were most prevalent across all snail gut samples, and give insight into the core microbiome. We also conducted a multilevel analysis of pattern (multipatt) using the “multpatt” function of the {indicspecies} package [30] to compare bacterial species between groups. The mulitpatt shows bacterial species that are significantly associated to treatment groups, or treatment group combinations.

To evaluate and visualize the taxonomic makeup of our treatment groups, we ran Kruskal-Wallis tests comparing relative abundances of bacterial families and genera for all snail guts using the “taxa_summary_by_sample_type” function in {MCToolsR}, which adjusted for multiple testing using Benjamini-Hochberg corrections (P_FDR_ < 0.05). We visualized the taxonomic compositions with the “plot_taxa_bars” function in {MCToolsR}. Family-level resolution was used for broad taxonomic visualization rather than genus-level to reduce the number of classifications reported and keep figures accessible for readers. For all other analyses, genus-level taxonomic resolution was used, rather than family-level.

## Results

### Sequencing results and taxonomic composition of gut bacterial community

The microbiome composition of *Oreohelix strigosa* in populations encompassing its native range proved to be highly diverse. In total, there were 5,278,630 reads sequenced, and 3,216,093 reads sequenced for only *O*. *strigosa* gut samples (2,062,537 reads belonged to soil and vegetation samples). The average number of reads per snail gut was 14,958.57± standard deviation (SD) 6579.79, with a maximum number of reads of 34,344 and a minimum number of 432 reads. The identified ASVs belonged to 85 unique phyla, 583 families, and 1,434 genera. There were 66,098 total ASVs identified in *O*. *strigosa* guts.

No ASVs were common to 100% of *O*. *strigosa* gut microbiome samples. There were three ASVs common to 90% of gut samples, these being ASV 1 (member of family Enterobacteriaceae), ASV 3 (member of family Sphingobacteriaceae) and ASV 9 (member of family Sphingomonadaceae). There were 11 ASVs common to 80% of gut samples, including ASVs 1, 2, 3, 5, 7, 8, 9 10, 14, 16, and 20, which comprised members of the bacterial families Enterobacteriaceae (two ASVs), Comamonadaceae, DEV007 (from order Verrucomicrobiales), Intrasporangiaceae, Micrococcaceae, Pseudonocardiaceae, Spirosomaceae, Sphingobacteriaceae, Sphingomonadaceae, and Trueperaceae.

### Geographic location and gut microbiome composition

#### Microbiome variation across the native range

**Fig 3:**
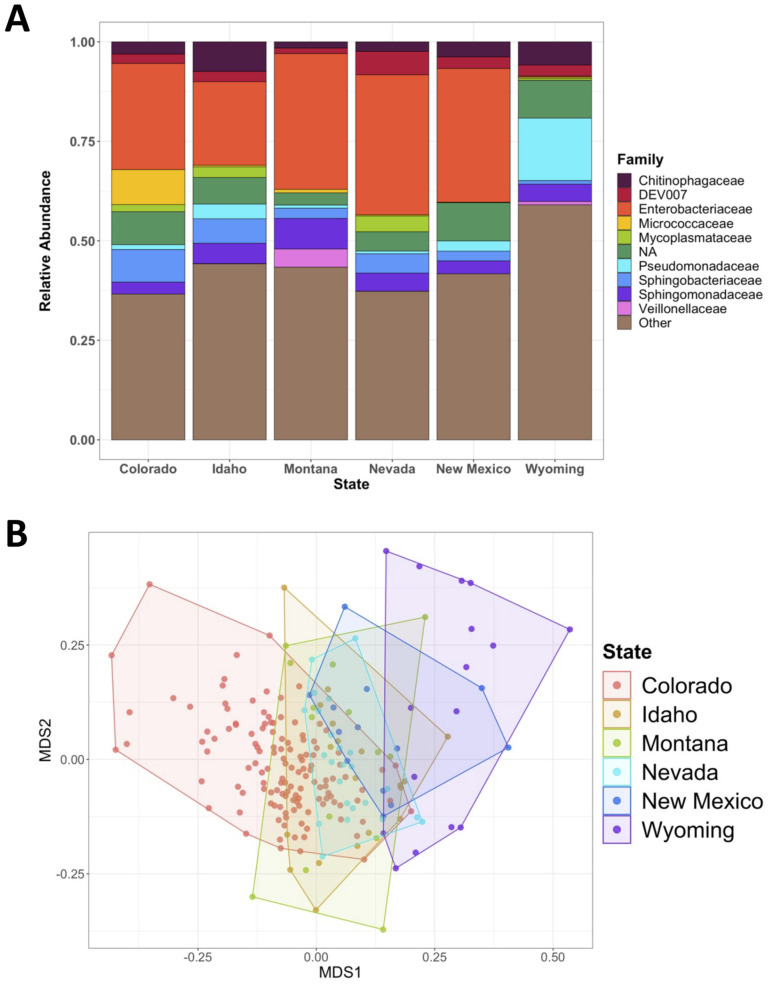
Gut microbiome differences by state: **(A)** Relative abundances of the top ten bacterial families contributing to each snail sample set collected from all sampled states **(B)** Non-metric multidimensional scaling analysis based on state of origin (PERMANOVA: *p*-value < 0.001, R^2^ = 0.09). NA refers to taxa that were unresolved at the family level, but were derived from a variety of orders. DEV007 refers to a family within the order Verrucomicrobiales.

There were ten ASVs found in every sampled state (ASV 1, 2, 7, 9, 10, 14, 16, 20, 25, 37). These included two members of bacterial families Enterobacteriaceae, two members of Sphingomonadaceae, and one member each of Comamonadaceae, DEV007 (from order Verrucomicrobiales), Intrasporangiaceae, Nocardioidaceae, Pseudonocardiaceae, and Trueperaceae. Samples from the state of Wyoming showed significantly less overall richness and significantly lowered relative abundances of taxa from the family Enterobacteriaceae compared with all other samples (Fig 3A). As there were multiple populations from Wyoming which were extracted over multiple days with samples with other states, we believe this is true variation in composition and richness, and not the effect of contamination or sampling bias.

We assessed how microbiome composition changed across *O*. *strigosa’s* native geographic range. There were significant differences in microbial community compositions based on geographic location. Looking broadly at the state collected, 9% of the variation in gut community composition was explained just by the state the snail was collected from (PERMANOVA: R^2^ = 0.09, *p*-value < 0.001; Fig 3B). Exact location (or verbatim locality) rather than broadly state, explained 32% of the variation across microbial communities (PERMANOVA: R^2^ = 0.32, *p*-value < 0.001).

We also investigated how community composition varied by other available metadata metrics across all samples. The elevation of a snail population only explained around 1% of the variation in gut community composition across the native range (PERMANOVA: R^2^ = 0.01, *p*-value < 0.001), and the year the sample was collected explained around 3% of the variation (PERMANOVA: R^2^ = 0.03 *p*-value < 0.001).

A Mantel test showed that geographic distances and the microbial Bray-Curtis dissimilarities were significantly correlated (Mantel statistic r = 0.21, *p*-value < 0.001). As samples became physically more separated, their corresponding microbial communities become more dissimilar.

In the most northern (Montana) and southern (New Mexico) localities sampled, there were significantly different relative abundances of some of the top taxa, including higher relative abundances of ASVs 2 (*Butiauxella* sp.) (P_FDR_ < 0.001) and 50 (unidentified member of family Enterobacteriaceae) (P_FDR_ < 0.001) in Montana; while New Mexico had significantly higher abundances of ASVs 1 (*Klebsiella* sp.) (P_FDR_ < 0.001) and 4 (*Raoultella* sp.) (P_FDR_ < 0.001).

#### Microbiome variation across the Colorado front range

**Fig 4:**
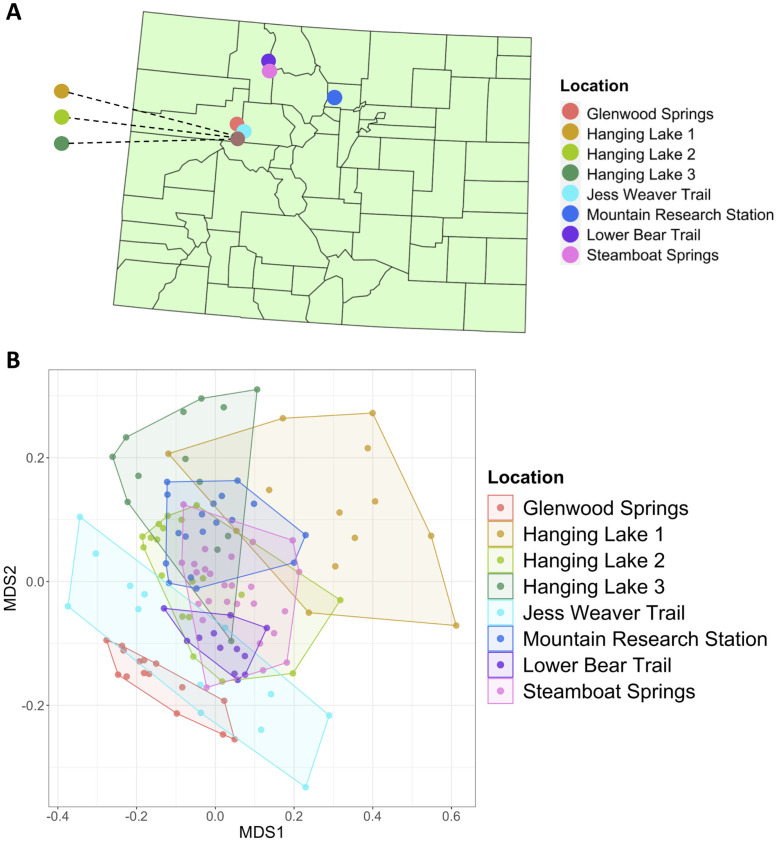
Gut microbiome differences within Colorado by locality: **(A)** Map of all *O*. *strigosa* sampling locations in Colorado **(B)** Non-metric multidimensional scaling analysis based on collecting locality (PERMANOVA: *p*-value < 0.001, R^2^ = 0.31).

Similar to the results of all snails from the native range, 31% of gut microbiome variation in snails across the Colorado Front Range (Fig 4A) was explained by locality (PERMANOVA: R^2^ = 0.31, *p*-value < 0.001; Fig 4B).

The elevation explained around 1.5% of the variation in gut community composition across the Coloradan samples (PERMANOVA: R^2^ = 0.015, *p*-value < 0.001), and there was no effect of year, as all Coloradan samples were collected in the same year. Summarized results of all conducted PERMANOVA analyses are reported in Table 2.

**Table 2:** Differing factors explain gut microbiome variation across *O*. *strigosa* samples collected across their native range and within Colorado. R^2^ and *p*-values calculated using Permutational Multivariate Analysis of Variance, with left column as the explanatory variable. Gut microbiome composition is significantly associated with all variables, but most explained by location.

Explanatory Variable	R^2^	*p*-value
***All Snail Gut Samples***		
State	0.088	<0.001
Location	0.324	<0.001
Elevation	0.015	<0.001
Year	0.038	<0.001
***Colorado Snail Gut Samples***		
Location	0.305	<0.001
Elevation	0.015	<0.001
Habitat Type	0.107	<0.001
Habitat Complexity	0.063	<0.001
Human Impact	0.094	<0.001

### Environmental factors and gut microbiome variation

We used the environmental metadata collected from local Coloradan field sites to investigate their explanatory effect on gut microbiomes. Collectively, environmental factors including habitat type, habitat complexity, and human impact level, explain about 27% of the variation across *O*. *strigosa* gut microbiomes (see below for detailed discussion).

#### Environmental sources of bacteria

**Fig 5:**
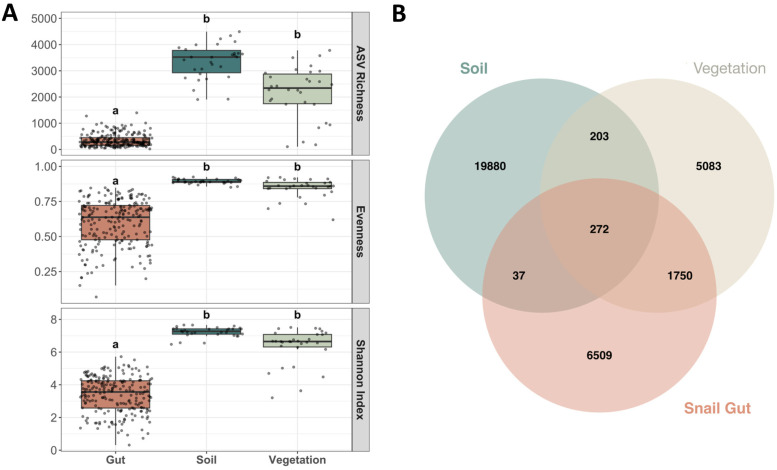
**(A)** Diversity boxplots showing differences in species richness, species evenness, and Shannon Index of snail gut, soil and vegetation microbiomes. Jitter shows distribution of samples. Letters above bars indicate significant differences. **(B)** Three-way Venn diagram of the microbial ASV composition in the microbial communities of snail gut, soil, and vegetation groups.

Snail gut microbiomes showed more similarity to vegetation microbiomes than soil microbiomes. Soil and vegetation samples had significantly higher bacterial richness, evenness, and Shannon Index than soil gut samples (Fig 5A, Table 3).

**Table 3:** Alpha-diversity metrics significantly differ by sample types (gut, soil, or vegetation) based on the results of Kruskal-Wallis tests. Data are reported as mean ± standard deviation.

	Snail Gut	Soil	Vegetation	*p*-value
**ASV Richness**	351.67 ± 246.08	3,354.97 ± 667.86	2,174.03 ± 989.18	<0.001
**Evenness**	0.60 ± 0.16	0.89 ± 0.02	0.84 ± 0.07	<0.001
**Shannon Index**	3.39 ± 1.10	7.23 ± 0.31	6.34 ± 1.12	<0.001

Though both soil and vegetation samples had significantly higher microbial richness than gut samples, vegetation samples shared 28% of their ASVs with snail guts while soil samples shared only 1.5% of their ASVs. The snail gut microbiome shared over 1,700 more ASVs with the vegetation microbiome (2,022 total ASVs shared) than with the soil microbiome (309 total ASVs shared) (Fig 5B). A multilevel pattern of analysis showed that only three bacterial taxa were specifically associated to both snail gut and soil microbiomes, while 47 were specifically associated to snail gut and vegetation microbiome.

Taxonomically, there were significant differences between snail gut, soil, and vegetation microbiome. At the phylum level, soil samples had significantly greater relative abundances of Planctomycetota, Acidobacteriota, and Verrucomicrobiota taxa (all P_FDR_ < 0.001) than both vegetation and gut samples. At the family level, soil samples had significantly greater abundances of Chthoniobacteraceae and Vicinamibacteraceae taxa (all P_FDR_ < 0.001) than both snail gut and vegetation samples. There were significantly greater relative abundances of Enterobacteriaceae taxa (P_FDR_ < 0.001) in snail gut samples (22%) compared with 0.03% in soils and 0.05% in vegetation. Relative abundances of Sphingobacteriaceae taxa in snail gut samples were similar to abundances in vegetation samples, and significantly higher than abundances in soil samples (P_FDR_ < 0.001).

#### Habitat type and gut microbiome composition

**Fig 6:**
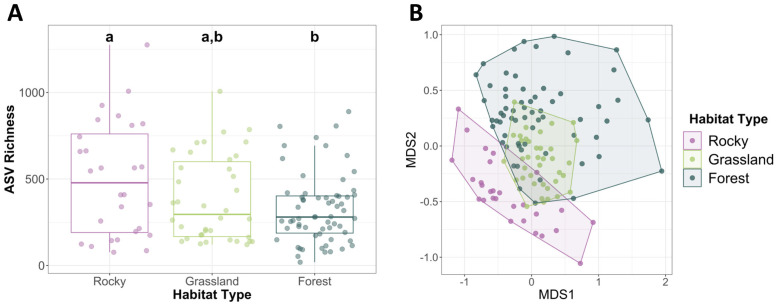
**(A)** Average microbial richness in varying habitat types. Rocky habitats had significantly higher microbial richness than forested habitats. Letters indicate significant differences. Error bars indicate standard error. **(B)** Non-metric multidimensional scaling analysis by site habitat type (PERMANOVA: *p*-value < 0.001, R^2^ = 0.11).

When habitats were categorized by the type of cover present, they fell into three categories—exposed rocks (rocky), grasslands, and forests. Microbial richness was highest in the rocky habitats, and lowest in the forested habitats, with grassland habitat richness being non-significantly different than either rock habitats or forested habitats (Fig 6A). Habitat type as a factor explained about 11% of the microbial variation across samples (PERMANOVA: R^2^ = 0.11, *p*-value < 0.001; Fig 6B)—more than any of the other environmental factors. A multilevel pattern of analysis showed that there were 773 bacterial taxa significantly associated to rocky habitat microbiome communities, 348 bacterial taxa significantly associated to grassland habitat microbiome communities, and 114 bacterial taxa significantly associated to forested habitat communities.

#### Habitat complexity and gut microbiome composition

**Fig 7:**
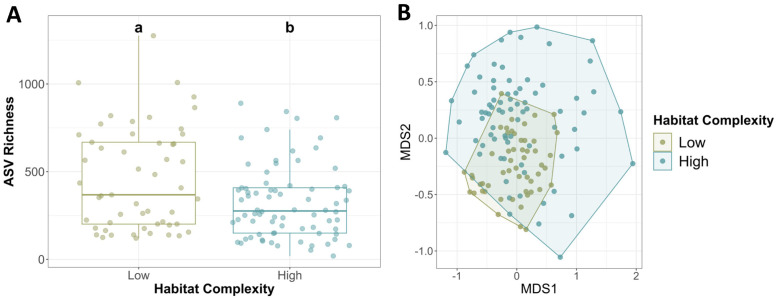
**(A)** Average microbial richness in varying habitat complexities. Low habitat complexity had significantly higher richness than high habitat complexity. Letters indicate significant differences. Error bars indicate standard error. **(B)** Non-metric multidimensional scaling analysis by site habitat complexity level (PERMANOVA: *p*-value < 0.001, R^2^ = 0.06).

Gut bacterial richness associated with high complexity habitat was significantly lower than that of low complexity habitat (Fig 7A). Habitat complexity as a factor explained about 6% of the microbial variation across samples (PERMANOVA: R^2^ = 0.06, *p*-value < 0.001; Fig 7B)—the lowest percentage explained of the environmental factors investigated. A multilevel pattern of analysis showed that there were no bacterial taxa significantly associated to either high or low habitat complexity microbiome communities.

#### Human impact and gut microbiome composition

**Fig 8:**
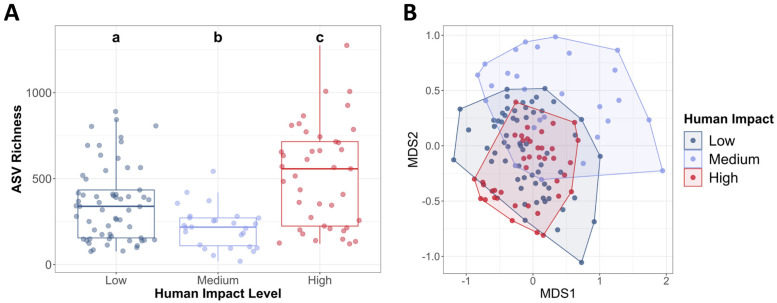
**(A)** Average microbial richness in varying levels of human impact. High human impact populations had significantly higher microbial richness lower levels. Letters indicate significant differences. Error bars indicate standard error. **(B)** Non-metric multidimensional scaling analysis by site habitat type (PERMANOVA: *p*-value < 0.001, R^2^ = 0.09).

Gut microbial richness was highest in the high human impact locations, and lowest in the moderate human impact locations (Fig 8A). Human impact level explained about 9% of the microbial variation across samples (PERMANOVA: R^2^ = 0.09, p-value < 0.001; Fig 8B). A multilevel pattern of analysis showed that there were over 800 bacterial taxa significantly associated to high human impact microbiome communities, while low and medium microbiomes each had around 150 bacterial taxa significantly associated to their groups.

## Discussion

In this study, we characterized the gut microbiome of *O*. *strigosa* specimens across their entire native range. We used alpha and beta diversity metrics to assess the effects of geography on gut microbiome compositions. We also investigated how environmental factors potentially explain patterns seen at a large geographic scale. Our results show that *O*. *strigosa* gut microbiome is variable across the broad geographic range, with up to 32% of variation explained by the location collected. When narrowing in on just the Colorado Front Range, we still see a large amount of microbiome variation explained by location (31%). To further tease apart the causal mechanisms of the geographic patterns, we investigated several possible determinants of variation, including soil and vegetation composition, habitat complexity, habitat type, and human impact. We conclude that several small-scale environmental factors may be associated with the changes in *O*. *strigosa* gut microbiome composition.

### Geographic location and gut microbiome composition

Bacterial composition of the *O*. *strigosa* gut varied significantly across geographic localities. Other available metadata including elevation and year collected did not explain as much of the variability. As snail gut samples became geographically more separated, their corresponding microbial communities become more dissimilar. Therefore, geographic location is a driving factor in shaping *O*. *strigosa’s* gut microbiome community.

The taxonomic compositions of snail gut microbiomes across the native range were generally consistent with previous findings looking only at snails from Colorado [20, 21]. Out of the top five ASVs found in snail guts, the top four were all bacteria from family Enterobacteriaceae, and the fifth was a member of family Sphingobacteriaceae (from genus *Pedobacter*). Results from previous work examining *O*. *strigosa* across life stage, time in preservation, and location similarly show the core microbiome primarily consists of members of Enterobacteriaceae and Sphingobacteriaceae [20, 21], which are hypothesized to aid in the degradation and fermentation of cellulose, hemicellulose, and lignin, all typical in this snail’s inferred diet [31]. Our finding further supports that these core microbial strains likely form obligate symbiotic relationships with *O*. *strigose*, as they are consistently found across a wide geographic range.

Similar studies investigating geographic effects on animal gut microbiome corroborate our findings. Geography has been observed to determine variations in mammalian gut microbiome composition [10]. For example, the gut microbiome of lowland gorillas is largely explained by geographic range, as is the fecal microbiome of the takahē bird [11, 32]. Similarly, gut microbiomes vary geographically in zebrafish, even explaining more variation than domestication does [33]. However, within these studies there are often other driving factors intertwined with physical location that may explain the patterns of microbiome composition across geography. For instance, fluctuations in microbiome composition in macaques across a broad geographic range may also be tied to elevation differences, with higher populations’ microbiomes having some adaptations to high-altitude environments [18]. Gorilla gut microbiomes were closely tied to geography, but also to the corresponding food available at each sampled site [32]. Therefore, physical distance may not be the only factor driving microbiome compositional changes across *O*. *strigosa’s* native range.

### Environmental factors and gut microbiome variation

#### Microbiome variation across the Colorado front range

To further elucidate how geographic location impacts gut microbiome composition, we narrowed in on samples originating from the state of Colorado. As with all the samples across the native range, there was a very low amount of variability explained by elevation, and no effect of preservation.

Animals of the same species that live geographically closer tend to have gut microbiome compositions more similar than those that live further away [12, 18]. This pattern may be attributed to animals being exposed to the same local environment and similar resources, like food availability [10, 32].

Below, we discuss some of the potential causal mechanisms of the geographic variation seen in Colorado using metadata collected in the field. These metadata are hypothesized ecological mechanisms that may be contributing to the microbiome patterns, but are in no way definitively responsible. Further controlled experimental studies are needed to test what ecological factors are driving the significant changes found across our samples. However, these metadata could provide further insight into which variables may be most important to investigate in future studies.

#### Environmental sources of bacteria

*O*. *strigosa* are likely gaining more gut bacterial taxa exogenously from their habitat’s vegetation than from the soil, as snail gut microbiomes showed more similarity to vegetation microbiomes than soil microbiomes. There were taxonomic differences between the compositions of soil microbiomes compared to gut and vegetation microbiomes. Importantly, there were significantly greater relative abundances of Enterobacteriaceae taxa, which have previously been shown to be core members of the *O*. *strigosa* gut microbiome, in snail gut samples compared with soil and vegetation samples. Relative abundances of another core family, Sphingobacteriaceae, in snail gut samples were similar to abundances in vegetation samples, and significantly higher than abundances in soil samples.

The inferred diet of *O*. *strigosa* of decaying wood and leaf litter supports that vegetation is more likely where snails exogenously uptake bacteria [34]. As *O*. *strigosa* preferentially feeds on decaying lignocellulosic matter rather than fresh vegetation, this discrepancy in our collecting method of mainly fresh vegetation may be why there were not greater similarities between the abundances of cellulolytic bacteria in snail gut samples and vegetation samples [34].

Different snail populations interact with different plant communities, as such this could contribute to the observed microbiome differences between localities. In some invertebrate host/bacterial symbiont systems, microbes are transmitted horizontally through plant-based diets. Many other land snails use a generalist feeding strategy, and thus have evolved unique gut microbiomes to efficiently breakdown and use a variety of tough, cellulolytic, vegetative materials for their own nutrition and growth [35]. Some terrestrial snails are known to augment their gut microflora through horizontal transmission via eating of plants and soils [31]. The gut microbiome of the giant African land snail is so intimately tied to diet that it can be modified by various plant species diets [36]. Beyond mollusks, phytophagous insects gain endosymbionts such as *Rickettsia*, *Wolbachia*, and *Cardinium* horizontally via plants [37]. Members of Lepidoptera may form their gut microbiomes through a combination of horizontal transmission via plants, and vertical transmission in their egg stage [38].

In other animal species, gut bacteria are transmitted horizontally from host to host. For instance, plateau pikas eat yak feces when food is scarce to gain beneficial bacterial symbionts [39]. While the authors do not know of any such reciprocal interaction between *O*. *strigosa* and another host species, *O*. *strigosa* are detritivores, and it is possible other decaying matter besides vegetative matter make up a part of their diet and could contribute to their gut microbiome.

Snails may be gaining bacterial symbionts endogenously, rather than exogenously, which might amplify the effects of isolation by distance in shaping the gut microbiome. Several other mollusks have been hypothesized or shown to pass down bacterial symbionts though vertical transmission [6]. *O*. *strigosa* is inferred to receive some of its microbes vertically, from parent to offspring [20]. Receiving important microbial taxa directly from the parent may confer some type of evolutionary advantage, allowing the ovoviviparous offspring of *O*. *strigosa* access to necessary symbionts to help them process complex, lignocellulosic molecules as soon as they are born. Previous work has shown that members of Enterobacteriaceae, which were in significantly lower abundances in soil and vegetation samples compared to gut samples, are present in both adult and fetal, unborn *O*. *strigosa* gut microbiomes, indicating these may be some of the taxa that snails receive vertically rather than horizontally [20]. Importantly, many of the previously identified core microbiome taxa [20, 21] were found only in high abundances in snail gut samples, and not in the soil or vegetation samples. Since the core bacteria may be directly passed down from parent to offspring, this could augment the effects of geography on snail gut microbiome compositions, as snail populations separated by great distances don’t typically interact.

#### Habitat type and gut microbiome composition

Habitat type as a factor explained the snail gut microbial variation more than any of the other environmental factors. In other animal hosts, changes in habitat greatly shapes gut microbiome composition. Growing evidence shows that organisms, including their gut microbiomes, can acclimate or adapt to different habitats [40], with the hypothesis being that gut microbial compositions are plastic, and can change with host physiological changes [41, 42]. When exposed to habitat exchanges (for instance, lake habitat to river habitat exchange), prawn showed significant differences in gut microbial compositions in as little as six months [40]. Likewise, tench gut microbiomes are more strongly shaped by environment, *i*.*e*., whether the fish was in a semi-intensive pond versus a lake, than other factors like seasonality [43]. Tasmanian devils, giant pandas, red pandas, and koalas all show markedly different and largely disrupted gut microbiome compositions in captive habitats versus their wild, natural habitats [10, 44–46].

Microbiomes from different habitat types differed in both richness and dominant bacterial taxa. We found significant differences in microbial richness between *O*. *strigose* habitat types, with the most ASVs found in rocky habitats and the least in forested habitats (Fig 6A). The top taxa present in each habitat type also varied greatly. Top bacteria found in the rocky habitats included members of bacterial families *Fluviicola*, *Pantoea*, *Taibaiella*, and *Truepera*. Members of these families are found in other studies to be specific to areas where limestone rock is dominant [47–49]. Importantly, limestone availability is one of the key factors for *Oreohelix* in building their calcium-rich shells [19]. *Oreohelix* populations in rocky habitats may be more directly exposed to limestone, and may be ingesting the bacteria that preferentially exist on limestone more than snails from grasslands or forests. Bacteria more common to grasslands included members of *Chryseobacterium* and *Mycoplasma*. Members of *Chryseobacterium* are inferred to inhibit plant pathogenic fungi in grassland habitats [50], and one member of *Mycoplasma* is a pathogen common to grassland arthropods, like grasshoppers [51]. Bacteria more common in forested habitats are lignin-degrading bacteria including members of genera *Raoultella*, *Spirosoma*, and family Micrococcaceae, which help to break down tough, woody matter typical of forest environments [52]. *Oreohelix* in wooded habitats may tend to consume more woody matter than those in grasslands or rocky habitats, reflected in their top bacterial taxa being lignin-degrading.

#### Habitat complexity and gut microbiome composition

As habitats became more complex, bacterial richness significantly decreased in the snanil guts. Our results represent a counterintuitive response of microbiome richness to high and low habitat complexities, as much of the literature shows that microbial richness increases with habitat complexity. For example, increased prey diversity significantly increases the gut bacterial diversity of predatory insects [53]. A more diverse diet and higher habitat complexity are associated with a healthier, more diverse gut microbiome in ruminant mammals [54]. Gut microbial diversity and richness in black howler monkeys decrease when diet and habitat complexity are also less diverse [16]. In *O*. *strigosa*, we see the opposite trend, with increased bacterial richness in the less complex habitats. We believe this trend is not due to sampling bias; as there were multiple, independent sites that contained high and low habitat complexity. Low habitat complexity could represent a less ideal environment for the snails. They may need to keep a diverse suite of microorganisms to survive, as they are less available from the habitats. Additionally, not all animal species’ microbiomes respond the same way to stress, in the form of lowered habitat complexity or others. For example, frogs, cranes, salamanders, and lizards exhibit increased bacterial richness and diversity in captive environments [55–58].

In this study, habitat complexity and human impact were often tied to one another, and both factors showed counterintuitive responses to bacterial richness. Those habitats classified as low habitat complexity usually had high human impact, and high habitat complexity sites had medium or low human impact. As microbial richness increased significantly in the most disturbed habitats (low habitat complexity, high human impact), disturbed snails may be showing signs of dysbiosis and a lack of homeostasis in their gut microbiome. For example, microbiomes found in heat-stressed corals showed increased microbial diversity, with higher measures of both alpha- and beta- diversity in the heat-stressed treatment than in controls [59]. Ocean acidification can also increase microbiome variability in sea sponges [60]. Similarly, populations from more disturbed sites had much higher richness, and numbers of bacterial taxa specific to their environments, compared with less disturbed sites. This may be due to the host’s inability to regulate which incoming bacteria from the surrounding environment will be accepted or rejected; this can thus result in a higher number of bacterial taxa than the host would normally allow [61]. Anthropogenically-induced stressors, which may include human presence and land-use change, may cause the microbiomes of disturbed snails to take on a wider range of possible configurations than their undisturbed counterparts.

Additionally, it is possible that members of *Oreohelix* may not be generalist feeders and may instead prefer to consume only certain vegetation. As such, habitat complexity may not have much of an impact on microbiome composition because snails largely depend on only certain plants in their diet. For instance, *Oreohelix* populations are commonly found co-existing with quaking aspen (*Populus tremuloides*). Even a single meal can increase the richness and alter the community composition of gut bacteria in invertebrates like lady beetles, indicating that the gut microbiome composition may be largely impacted by the exact time a sample was collected [53] Future studies should use laboratory experiments to confirm how drastically the *Oreohelix* gut microbiome can change meal to meal. There may also be other biological interactions at play in these sites causing variation in microbiome richness, like predation, parasitism, or pathogens, that were not captured in this study.

#### Human impact and gut microbiome composition

Similar to the above discussion, human impact level had a counterintuitive effect on microbial richness. In many other animal species, increased anthropogenic distances reduce the diversity and richness of the microbiome. Gut microbiome plasticity may be an important mechanism by which animals can adapt to environmental change, especially anthropogenically caused changes [62, 63]. Land-use change may shift food availability, quality, and overall diet for local species [63]. For example, urbanization of natural habitat reduces lower gut bacterial richness and alters the community composition in house sparrows [64]. However, other birds like white-crowned sparrows and ground finches show opposite trends, with more diverse gut microbiomes in urban populations, likely due to a more diverse diet [65, 66]. Likewise, water dragon gut microbiomes show increased microbial diversity in urbanized habitats, suggesting a shift to a more diverse diet in these urban habitats [62]. As increased gut microbial diversity is often thought to reflect a more diverse diet [67–69], increased richness in snails from habitats with higher human impact may be due to increased diversity of their diets. Human disturbances, like urbanization, often bring in more exotic, non-native plant species to a habitat [62]. Snails in high human impact areas may be feeding on these novel plant sources, causing an increase in rarer bacterial taxa. Snails in heavily impacted habitats may be urban exploiters and use their diverse and responsive gut microbial community as important adaptive mechanism to exploit novel urban environments and new food sources.

As stated earlier, levels of habitat complexity and human impact in a given site were often related to one another, and both factors showed counterintuitive responses to bacterial richness. Therefore, many of the same explanations for the counterintuitive microbial patterns in low habitat complexity sites may also be at play in influencing the high human impact sites.

## Conclusion

*Oreohelix strigosa* gut microbial composition changes with broad geographic disparities, for example, by the state in which the snail was collected. More specifically, microbial compositions differ greatly even across collection sites within states and regions. Ecological factors that vary within locations, like habitat complexity, habitat type, and human impact, can alter microbial composition and richness. Snail microbiomes may be responding to stressful factors, like increased human impact and lowered habitat complexity, in a counterintuitive way. All in all, snail gut microbiome compositions are not static—they shift with changing environments.

As land snails, particularly narrow-range endemic species, are among the most threatened animals on Earth, there are significant conservation implications for this work. An estimated 54% of North American land snails are threatened with extinction [70]. It has been established that other species of the *Oreohelix* genus (e.g., Black Hills Mountainsnail [*Oreohelix cooperi*]) are already being listed as ‘threatened’ at the state level, and local collectors have observed that the Rocky Mountainsnail is rapidly declining (while once common and widespread across the Colorado Front Range in the early 1900s, few populations remain) [71, 72]. Many populations exist only within certain geographically isolated rocky outcrops or canyons, which may in turn make their gut microbiomes equally as specialized [73]. For such species and subspecies, captive-breeding programs may be important for ensuring species survival, by supplementing wild populations or creating “backup” populations. There is concerning potential for captively bred animals, like snails, or significantly disturbed populations to lose key components of their wild microbiome that are essential to host function, which could result in failing conservation programs unless wild microbiomes are characterized broadly and early.

Overall, exploration of the biogeography of the gut microbiome composition is vital baseline information for future studies of *O*. *strigosa*, and this study may provide guidance to conduct similar microbiome surveys of other animal species with wide geographic distributions. This work will also help facilitate finer-scale studies which aid in the management and conservation of this prolific snail species across its native range.

## Supporting information

S1 Table Metadata of all specimens used in this study. (XLSX)
